# Seroprevalence of Tick-Borne Infections in Horses from Northern Italy

**DOI:** 10.3390/ani12080999

**Published:** 2022-04-12

**Authors:** Luca Villa, Alessia Libera Gazzonis, Carolina Allievi, Claudia De Maria, Maria Flaminia Persichetti, Giulia Caracappa, Sergio Aurelio Zanzani, Maria Teresa Manfredi

**Affiliations:** 1 Department of Veterinary Medicine and Animal Sciences, Università Degli Studi di Milano, Via Dell’ Università 6, 26900 Lodi, Italy; luca.villa@unimi.it (L.V.); alessia.gazzonis@unimi.it (A.L.G.); carolina.allievi@unimi.it (C.A.); mariateresa.manfredi@unimi.it (M.T.M.); 2 Istituto Zooprofilattico Sperimentale Della Sicilia “A. Mirri”, Via Gino Marinuzzi 3, 90100 Palermo, Italy; cdemariavet@gmail.com (C.D.M.); mfpersichetti@gmail.com (M.F.P.); giulia.caracappa@gmail.com (G.C.)

**Keywords:** *Anaplasma phagocytophilum*, *Babesia caballi*, *Theileria equi*, Rickettsiales, protozoa, piroplasmosis, granulocytosis, horse

## Abstract

**Simple Summary:**

Equine vector-borne diseases, which include equine granulocytic anaplasmosis (EGA) and equine piroplasmosis (EP), are caused by several pathogens transmitted to horses by ticks. Considering the spread of equine vector-borne diseases in Italy and worldwide, a study was planned to investigate the seroprevalence of *Anaplasma phagocytophilum* and the other two selected pathogens, *Theileria equi* and *Babesia caballi*, in northern Italy. Data obtained from the present study demonstrated a high seroprevalence for all the three surveyed tick-borne pathogens and emphasized the importance of establishing control programs with the adoption of certain practices, including tick control, correct horses management, serological screening, regular usage of long-lasting acaricides and proper treatment of positive and clinically infected animals.

**Abstract:**

Tick-borne diseases in horses are considered an emergent problem worldwide; the geographical redistribution of ticks, due to climatic and ecological changes, and the movements of infected horses between different nations play important roles in the spread of tick-borne diseases affecting these hosts. In this study, a survey was planned to estimate the seroprevalence of the Gram-negative bacterium *Anaplasma phagocytophilum* and the piroplasmid protozoa *Babesia caballi* and *Theileria equi* in Italian horses, as well as to evaluate possible risk factors associated with seropositive cases. Serum samples from 261 horses reared in northern Italy were collected and analyzed by indirect immunofluorescence antibody test for the detection of *A. phagocytophilum-*, *B. caballi-* and *T. equi*-specific antibodies. The overall seroprevalence to at least one of the investigated pathogens was 51%; sixty-one horses were seropositive to *A. phagocytophilum* (23.4%), forty-nine to *B. caballi* and the same number to *T. equi* (18.8% each). Seropositivity for more than one of the investigated agents was detected in thirty-two horses and the most common co-infection was observed between *B. caballi* and *T. equi* (5.7%). A significant risk factor for all the three pathogens was the elevation above sea level; indeed, the risk of infection was higher with an increase and decrease in elevation for *A. phagocytophilum* and for *B. caballi* and *T. equi*, respectively. Tick control in horses is strongly recommended considering the high seroprevalence values of transmitted pathogens.

## 1. Introduction

Equine vector-borne diseases are caused by several pathogens transmitted to horses by arthropods, particularly ticks. These ubiquitous parasites can live in a variety of environments, where climatic conditions allow their survival and development. Due to global warming and ecological changes, which have increased wildlife populations, ticks are expanding in non-endemic countries. In addition, the high plasticity of ticks allowed their adaptation to a wide host range and the lengthening of the seasonal transmission of tick-borne pathogens (TBPs).

Among these, *Anaplasma phagocytophilum* and the piroplasmid protozoa are important agents of tick-borne diseases in horses worldwide [1,2]. *A. phagocytophilum*, a Gram-negative intracellular obligate bacterium, infects granulocytic cells, mainly neutrophils and eosinophils; its principal vector in Europe, including Italy, is *Ixodes ricinus* [3]. A wide range of both domestic and wild mammals can develop granulocytic anaplasmosis, caused by members of the *A. phagocytophilum* complex, which includes the agents of tick-borne fever in ruminants (TBF), equine granulocytic anaplasmosis (EGA) and canine granulocytic anaplasmosis (CGA) and of human granulocytic anaplasmosis (HGA). In horses, EGA is characterized by aspecific clinical signs, including lethargy, depression, fever, limb edema, hemorrhagic petechiae, ataxia and thrombocytopenia [1,4].

*Babesia caballi* and *Theileria equi* are the etiological agents of equine piroplasmosis (EP) and parasitize erythrocytes. In Europe, they are mainly transmitted by five genera of hard ticks: *Dermacentor*, *Hemaphysalis*, *Hyalomma*, *Ixodes* and *Rhipicephalus*; the main tick species now recognized as competent vectors are *D. marginatus*, *D. reticulatus*, *I. ricinus*, *R. bursa* and *R. sanguineus* [5,6,7]. *B. caballi* is transmitted in ticks via both transstadial and transovarial ways, while in contrast *T. equi* is transmitted only trans-stadially [6]. These two hemoprotozoa are endemic in tropical and temperate areas, such as in several southern European countries, including Italy, and cause important economic losses due to treatment costs, abortions and loss of activity, and in some cases also death [8,9]. Clinical signs of EP are comparable to those of EGA: fever, ventral edema, icteric sclera, pale mucous membranes, anemia, hemoglobinuria, bilirubinuria, weakness and lethargy. EP can occur in acute, sub-acute or chronic forms, and after the acute phase horses may remain seropositive, becoming silent carriers of the protozoa [1,8,9]. Seropositivity to these pathogens involves the restriction of the movement of horses among countries; indeed, serological tests for EP are mandatory for international movement, since only seronegative horses for both *T. equi* and *B. caballi* can be imported to the United States, Canada, Australia and Japan [10].

EGA and EP can be diagnosed using direct and indirect methods. Intra-erythrocyte piroplasms and *Anaplasma* spp. morulae can be directly detected via microscopic examination of Giemsa-stained peripheral blood or buffy coat smears. However, when the parasitemia is mild or low, parasite detection is difficult. The molecular determination of TBP DNA is a sensitive and specific diagnostic approach, but it is only useful in early infection. Further, several indirect methods based on antibody detection in sera are available to assess infection during the latent period characterized by microscopically and molecularly undetectable parasitemia, and these tests are also useful for large-scale studies [2,4,11].

In Italy, mainly in the northern areas, information regarding the epidemiology of TBPs in horses is limited, especially for *A. phagocytophilum*. Indeed, most of the published data are related to serosurveys carried out in central Italy or Sicily [1,12,13,14,15].

Considering the importance of these tick-borne diseases for horses’ health and welfare, as well as the zoonotic aspects of anaplasmosis, a study was planned to investigate the seroprevalence of *A. phagocytophilum* and the two other selected pathogens, *T. equi* and *B. caballi*, in northern Italy, evaluating possible risk factors associated with seropositive cases to contribute to the surveillance of these TBPs and to control their diffusion among horses and humans. Serological studies can include many animals and allow the large-scale assessment of exposure to pathogens, even if to define an animal as infected it is necessary to associate the serology to the direct detection of the parasite using molecular methods. Indeed, PCR assays are very useful in cases of early infection when IgG antibodies have not yet been produced or are not detectable and to detect persistent infections [1,4].

## 2. Materials and Methods

Considering the population of horses in Lombardy and Piedmont of 78,490 animals (National Zootechnical Database, https://www.vetinfo.sanita.it/; accessed on 1 February 2016), a minimum sample size of 246 horses was estimated, as previously described for an epidemiological survey on protozoa infections in equids [16]. Overall, 261 horses (*Equus caballus*) from 35 stables located in Lombardy and Piedmont regions at different altitudes (from 60 to 1500 m above sea level (asl)) were included. None of the horses showed clinical symptoms. From April 2016 to March 2017, blood samples were collected through jugular vein puncture, using a Vacutainer^®^ sterile collection system, then refrigerated in tubes without anticoagulants during the transport to the laboratory. Once in the laboratory, sera were separated by centrifugation (2120× *g*, 15 min) and then stored at −20 °C until serological analyses. Subsequently, samples were analyzed with a commercial indirect immunofluorescence antibody assay (IFAT IgG Kit, Fuller Laboratories, Fullerton, CA, USA) for the detection of antibodies against *A. phagocytophilum*, *T. equi* and *B. caballi*, using a screening dilution rate of 1:80 in a phosphate-buffered saline solution (pH 7.2), as described in the manufacturer’s protocol.

For each sampled horse, data on the following variables were collected to identify possible risk factors: age (in years, ≤30), destination (companion/meat production), gender (male, female), stable elevation (in meters, asl), outdoor housing (yes/no, only box), stable size (small/large, ≥40 animals). Firstly, univariate binary logistic regression analysis was performed to determine factors that could be predictors of seropositivity for the three selected pathogens. In a second step, the variables showing *p*-values < 0.1 were entered into a multivariate model developed by backward elimination until all remaining variables were significant (*p* < 0.05). Statistical analysis was conducted using SPSS software (Statistical Package for Social Science, IBM SPSS Statistics for Windows, Version 26.0., Chicago, IL, USA).

**Figure 1:**
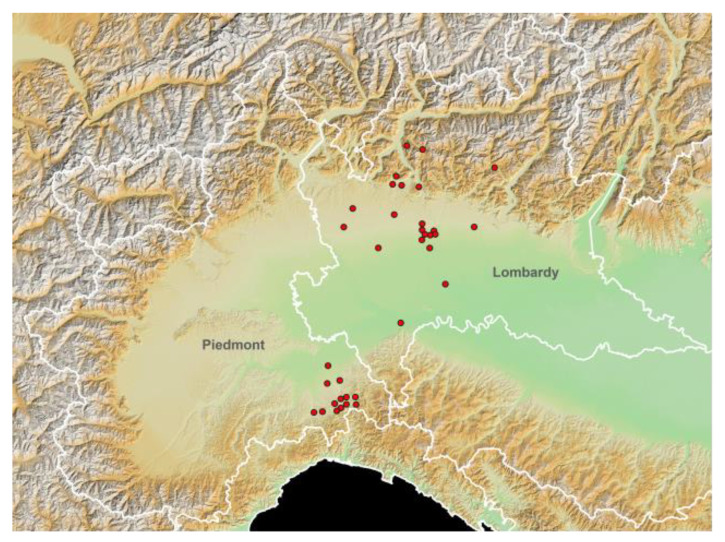
Study area elevation map (European Digital Elevation Model (EU-DEM), version 1.0; https://land.copernicus.eu/imagery-in-situ/eu-dem/eu-dem-v1-0-and-derived-products/eu-dem-v1.0?tab=metadata#:~:text=http%3A//land.copernicus.eu/pan-european/satellite-derived-products/eu-dem/eu-dem-v1-0-and-derived-products/eu-dem-v1.0/view; accessed on 15 November 2021) and spatial distribution of the tested horse stables (red dots). White lines: Italian regional boundaries.

The spatial distribution of the different stables, according to the different elevations, is represented in Figure 1 using the European Digital Elevation Model (EU-DEM, version 1.0).

## 3. Results and Discussion

In this study, an overall seroprevalence of 51% was estimated, with 133 out of 261 horses seropositive to at least one of the three investigated TBPs (Table 1).

The most common co-infections, i.e., *B. caballi* and *T. equi*, both agents of equine piroplasmosis, were observed in 15 out of 261 horses (5.7%). Concerning *A. phagocytophilum*, the detected seroprevalence of 23.4% was in line with another study recently carried out in central Italy reporting a seroprevalence of 22.75% [14], but higher than previous studies conducted in Italy with values ranging between 7.7% and 17.03% [1,4,13]. Some European countries have shown lower seropositivity varying from 6.5% to 13 % [2,17,18,19,20]. On the contrary, prevalence values comparable to those found in the present study were observed in southern Bulgaria and Denmark [21,22], whereas a higher seroprevalence (73%) was only reported in the Czech Republic [23].

The seroprevalence rates for both *B. caballi* and *T. equi* equaled 18.8%. In Italy, regarding *B. caballi*, previous studies reported values of 8.9% and 26% in horses from south and central Italy, respectively [1,9]. In other European countries, seroprevalence rates varied from endemic territories, such as Spain (21%) [24], France (12.9%) [25] and Portugal (11.1%) [2], to areas where values were lower, such as Greece (2.2%) [10] and Czech Republic (0.4%) [6]. Regarding *T. equi*, in northern Italy, a lower seroprevalence value of 13.3% was previously recorded [5], while the seropositivity rates rose to 39.8% and 41% in studies conducted in southern and central Italy, respectively [1,9]. In Europe, seroprevalence values varied from high positivity in France (58%) [25] and Spain (44%) [24], both considered endemic territories, to low values in Portugal (17.9%) [2], Greece (11%) [10] and Czech Republic (1.1%) [6]. Regarding *B. caballi*, the high variability in the seroprevalence could be related to the different distribution rates of ticks and infected animals, and also to the different diagnostic methods [1,10,24].

According to the univariate model (Table 2), the variables significantly associated with *A. phagocytophilum* seroprevalence were age, destination, elevation, and stable size. Indeed, the risk of infection decreased as age increased (OR = 0.936; 95% CI: 0.893–0.981), as previously observed by Laamari et al. (2020) [26].

However, increased seroprevalence as the age increased was reported in other studies, which was linked to a moderate increase in antibodies as the animals got older [21,23]. In relation to the geographical distribution of infected horses, an increase in the risk of infection was observed as the altitude increased; therefore, the odds for a horse being infected were 1.003 times higher for every one-meter increase in elevation. This relation could be linked to the large populations of *I. ricinus*, the main vector of *A. phagocytophilum*, in the surveyed geographical areas, where winters are temperate and summers are hot, with abundant rainfall throughout the year and relative humidity levels above 80%. Additionally, the whole territory is characterized by deciduous woodlands in which the high degree of humidity and the presence of reservoir hosts represent suitable conditions for the development of all life stages of the tick. Although *I. ricinus* was also described in lowland areas [27], the greater risk of exposition to *A. phagocytophilum* at higher altitudes was in line with studies that demonstrated the greater diffusion of the vector in hilly and mid-mountain areas [3]; these results could also be inferred from the human cases of Lyme disease that regularly occur in northern Italy [28].

Elevation was also associated with *B. caballi* and *T. equi* seroprevalence; indeed, the highest seroprevalence was found in the horses resident in stables located at a lower altitude, with the risk of infection increasing as the elevation decreased (OR = 0.998, 95% CI: 0.996–0.999 and OR = 0.997, 95% CI: 0.995–0.999 for *B. caballi* and *T. equi,* respectively). The weather and environment of the counties at lower elevation included in this study, characterized by the presence of wetlands, rice paddies and water meadows, provide an ideal habitat for the life cycle of the vectors of *B. caballi* and *T. equi* [29,30]. Other significant variables were the destination and stable size. Moreover, for *B. caballi*, age was not associated with infection risk as a result of the fact that infected horses eliminate the parasite in the blood within 4 years, with a progressive decrease in antibody titer, whereas horses infected by *T. equi* may remain as lifelong carriers [8,10,24]. Indeed, the univariate model confirmed that older animals had the highest risk of infection (OR = 1.053, 95% CI: 1.005–1.103) for *T. equi*. This could also be suggestive of an increase in antibodies with age, since older animals could have been exposed to ticks for a longer period than young animals [2,9,10,24,25].

In the final multivariable model (Table 3), obtained through backward elimination, the only significant variable for all three selected pathogens was elevation; indeed, the risk of infection increased as the elevation increased for *A. phagocytophilum*, while it decreased for *B. caballi* and *T. equi*. This result, as mentioned above in the univariate model, could reflect the geographical distribution and variety of ecological interactions of the ticks responsible for the transmission of these selected pathogens.

## 4. Conclusions

Horses living in northern Italy are to tick infestations and then to tick-borne diseases; indeed, data obtained from the present study demonstrated a high seroprevalence for all three surveyed TBPs. In particular, it was confirmed that horses are involved in the cycle of *A. phagocytophilum*, but further molecular analyses are required in order to identify areas where these three pathogens are actively circulating and to confirm the serological results. Surveillance is important both for *A. phagocytophilum*, due to its zoonotic impact, and for *B. caballi* and *T. equi*, for which the serological screening is mandatory for international movements. From the results obtained in this survey, control programs should be developed, with the adoption of practices including tick control, correct horse management, serological screening, regular usage of long-lasting acaricides and proper treatment of positive or clinically infected animals.

## Figures and Tables

**Table 1:** Seropositivity to *Anaplasma phagocytophilum*, *Theileria equi* and *Babesia caballi* and their co-infections with IFAT in 261 horses in northern Italy.

	*A. phagocytophilum*	*B. caballi*	*T. equi*	*A. phagocytophilum + B. caballi + T. equi*	*A. phagocytophilum + B. caballi*	*A. phagocytophilum* *+ T. equi*	*B. caballi * *+ T. equi*	Overall Seroprevalence
**Positive**	61	49	49	3	6	8	15	133
**Negative**	200	212	212	258	255	253	246	128
***p*%** (95% CI *)	23.4% (18.4–29)	18.8% (14.2–24)	18.8% (14.2–24)	1.1% (0.2–3.3)	2.3% (0.8–4.9)	3.1% (1.3–5.9)	5.7% (3.2–9.3)	51% (44.7–57.1)

Note: * 95% CI: Confidence interval.

**Table 2:** Potential risk factors associated with *Anaplasma phagocytophilum*, *Babesia caballi* and *Theileria equi* seropositivity in horses reared in northern Italy as assessed via univariate analysis. Test performed at 95% significance level. Note: *p*-values ≤ 0.1 considered significant.

Pathogen	Variable	Category	OR *	95% CI **	*p*-Value
*Anaplasma phagocytophilum*	Gender	Male	0.708	0.398–1.259	0.240
		Female	1		
	Age (years)	Continuous	0.936	0.893–0.981	**0.006**
		variable			
	Destination	Meat production	9.009	4.686–17.321	**<0.001**
		Companion (a)	1		
	Outdoor housing	No (only box)	0.532	0.226–1.267	0.155
		Yes (a)	1		
	Stable size	Large (≥40)	4.065	2.130–7.760	**<0.001**
		Small (a)	1		
	Elevation (m asl)	Continuous	1.003	1.002–1.004	**<0.001**
		variable			
*Babesia caballi*	Gender	Male	1.549	0.821–2.923	0.176
		Female	1		
	Age (years)	Continuous	1.029	0.982–1.078	0.236
		variable			
	Destination	Meat production	0.173	0.052–0.579	**0.004**
		Companion (a)	1		
	Outdoor housing	No (only box)	0.793	0.309–1.769	0.497
		Yes (a)	1		
	Stable size	Large (≥40)	0.401	0.208–0.773	**0.006**
		Small (a)	1		
	Elevation (m asl)	Continuous	0.998	0.996–0.999	**0.003**
		variable			
*Theileria equi*	Gender	Male	1.549	0.821–2.923	0.176
		Female	1		
	Age (years)	Continuous	1.053	1.005–1.103	**0.032**
		variable			
	Destination	Meat production	0.110	0.026–0.469	**0.003**
		Companion (a)	1		
	Outdoor housing	No (only box)	1.254	0.574–2.739	0.571
		Yes (a)	1		
	Stable size	Large (≥40)	0.756	0.405–1.410	0.379
		Small	1		
	Elevation (m asl)	Continuous	0.997	0.995–0.999	**0.001**
		Variable			

Statistically significant variables are indicated by bold type; * OR = odds ratio; ** 95% CI: confidence interval; a = baseline.

**Table 3:** Potential risk factors associated with *Anaplasma phagocytophilum*, *Babesia caballi* and *Theileria equi* seropositivity in horses reared in northern Italy by univariate analysis. Test performed at a 95% significance level. Note: *p*-values ≤ 0.1 considered significant.

Pathogen	Variable	Category	OR *	95% CI **	*p*-Value
*Anaplasma phagocytophilum*	Elevation	Continuous	1.003	1.002–1.004	**<0.001**
	(m asl)	variable			
*Babesia caballi*	Elevation	Continuous	0.998	0.996–0.999	**0.003**
	(m asl)	variable			
*Theileria equi*	Elevation	Continuous	0.997	0.995–0.999	**0.001**
	(m asl)	Variable			

Statistically significant variables are indicated by bold type. Note * OR = Odds ratio; ** 95% CI: Confidence interval.

## Data Availability

The datasets used and analyzed during the current study are available from the corresponding author on reasonable request.
